# Efficient targeted integration into the bovine Rosa26 locus using TALENs

**DOI:** 10.1038/s41598-018-28502-x

**Published:** 2018-07-10

**Authors:** Ming Wang, Zhaolin Sun, Zhiyuan Zou, Fangrong Ding, Ling Li, Haiping Wang, Chunjiang Zhao, Ning Li, Yunping Dai

**Affiliations:** 1 State Key Laboratory for Agrobiotechnology, College of Biological Sciences, China Agricultural University, Beijing, China; 2 College of Animal Science and Technology, China Agricultural University, Beijing, China

## Abstract

The genetic modification of cattle has many agricultural and biomedical applications. However, random integration often results in the unstable expression of transgenes and unpredictable phenotypes. Targeting genes to the “safe locus” and stably expressing foreign genes at a high level are desirable methods for overcoming these hurdles. The Rosa26 locus has been widely used to produce genetically modified animals in some species expressing transgenes at high and consistent levels. For the first time, we identified a bovine orthologue of the mouse Rosa26 locus through a genomic sequence homology analysis. According to 5′ rapid-amplification of cDNA ends (5′RACE), 3′ rapid-amplification of cDNA ends (3′RACE), reverse transcription PCR (RT-PCR) and quantitative PCR (Q-PCR) experiments, this locus encodes a long noncoding RNA (lncRNA) comprising two exons that is expressed ubiquitously and stably in different tissues. The bovine Rosa26 (bRosa26) locus appears to be highly amenable to transcription activator-like effector nucleases (TALENs)-mediated knock-in, and ubiquitous expression of enhanced green fluorescent protein (EGFP) inserted in the bRosa26 locus was observed in various stages, including cells, embryos, fetus and cattle. Finally, we created a valuable master bRosa26-EGFP fetal fibroblast cell line in which any gene of interest can be efficiently introduced and stably expressed using recombinase-mediated cassette exchange (RMCE). The new tools described here will be useful for a variety of studies using cattle.

## Introduction

Genetically modified cattle, which are generated using traditional transgene or gene targeting techniques, are important for improving the quality of milk^1^, for the recombination of pharmaceutically active proteins^2–6^ and for improving resistance to zoonotic diseases^7,8^. However, these methods are associated with random insertion, an uncontrollable copy number of the transgene and poor efficiency. Site-specific integration maybe a effective way overcome these hurdles^9,10^.

Recently, designer nucleases, such as zinc finger nucleases (ZFNs), transcription activator-like effector nucleases (TALENs) and CRISPR/Cas9, were reported to be suitable for genome editing in cattle and displayed high efficiency. Using this approach, exogenous DNA sequences are delivered into specific loci for comparative or subtractive gene function studies. However, the selection of a suitable “safe genomic acceptor site” is important for the wide application of this technology in gene transfer and to optimally design the transgene cassette to ensure robust expression without perturbing the transcription of nearby endogenous genes^11^.

Rosa26 is a safe-harbor locus broadly used for constitutive, ubiquitous gene expression in mice. Rosa26 was first isolated in 1991 in a gene-trap mutagenesis screen of murine ES cells^12^. Knock-in of a transgene into the Rosa26 locus enables locus-specific and copy number-controlled transgene expression and has been proved “safe” in mice because the transgene is adequately expressed without perturbing the endogenous gene structure or function^13^. Now, the insertion of a reporter or a toxin gene into the Rosa26 locus has been widely used to trace or ablate specific cell lineages^13^. Meanwhile, Recombination-mediated cassette exchange (RMCE) combined with the Rosa26 locus has important applications, such as predictable, stable and high-level expression not only for animal transgenesis but also for biotechnological purposes^14^. The Rosa26 locus is conserved in a variety of species, including humans^15^, rats^16^, pigs^17–19^ and rabbits^20^. However, researchers have not yet determined whether the Rosa26 locus exists in the bovine genome or whether this locus possesses similar characteristics as observed in other species.

In this study, we identified and characterized the bovine Rosa26 locus. We further conducted *in vivo* experiments to produce cattle with a knock-in at this locus to functionally validate its applicability. We also established a master bRosa26-EGFP cell line, which were used to perform RMCE mediated by TAT-Cre without a selective marker gene^21–23^. Our results add bRosa26 to the tool box of transgenic research in cattle. The availability of a safe-harbor locus in bovine is expected to improve the applications of genetically modified cattle.

## Results

### Identification of the bovine Rosa26 locus

**Figure 1:**
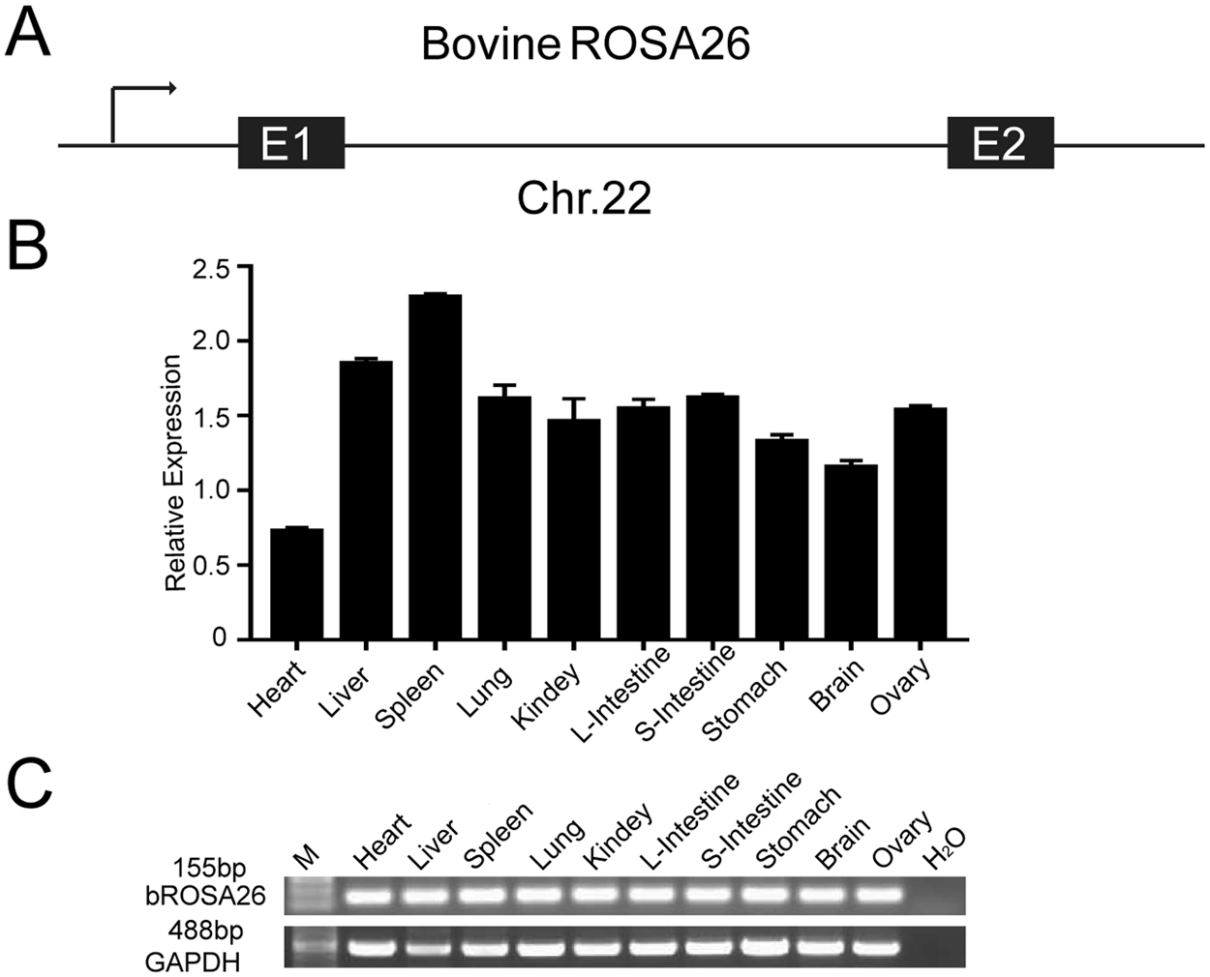
Identification of the bRosa26 locus. (**A**) Diagram of the bRosa26 locus on chromosome 22. (**B**) The expression of the bRosa26 lncRNA in various tissues relative to GAPDH was determined using Q-PCR. Error bars represent the mean ± SD. (**C**) The expression of the bRosa26 lncRNA in various tissues relative to GAPDH was analyzed using RT-PCR. For RT-PCR, the designed primers annealed to the bRosa26 sequence and amplified a correctly spliced product of 155 bp. GAPDH served as a control (488 bp).

The 1 kb mouse Rosa26 promoter and exon 1 sequences (NC_000072.6) were used as a template to search the Ensembl bovine database (UMD3.1.1). A highly conserved sequence (sequence similarity of 83%) on bovine chromosome 22 was identified as the putative bovine Rosa26 locus, based on the results from DNAMAN software. This region contained both the bovine Rosa26 locus and the neighboring genes that have also been identified in mice, rats, humans and pigs (Supplementary Fig. S1A). Alignments of the mouse, rat, pig, human and bovine Rosa26 promoter and exon 1 sequences showed high sequence conservation (>75%) among these species (Supplementary Fig. S1B). Bovine Rosa26 exon 1 was predicted by aligning the sequence to the mouse, rat, human and pig Rosa26 exon 1 sequences, and exhibited the greatest sequence conservation (>98%). These findings provided convincing evidence that the region on bovine chromosome 22 identified here represents the equivalent of the mouse Rosa26 locus and should thus be referred to as bovine Rosa26 (bRosa26). A screen of the Ensembl gene expression database for the bRosa26 locus failed to identify any expressed sequence tags (ESTs). Therefore, we designed primers aligned within predicted exon 1 to perform 5′ and 3′ rapid-amplification of cDNA ends (RACE) analyses and identified a noncoding RNA product of 377 bp transcribed from the bRosa26 locus that comprised two exons (Fig. 1A and Supplementary Fig. S1C). Reverse transcription (RT-PCR) and Quantitative PCR assays (Q-PCR) revealed this noncoding RNA expressing in a wide variety of adult tissues (Fig. 1B,C).

### Assessment of TALEN activity toward bRosa26 in bovine fetal fibroblasts

**Figure 2:**
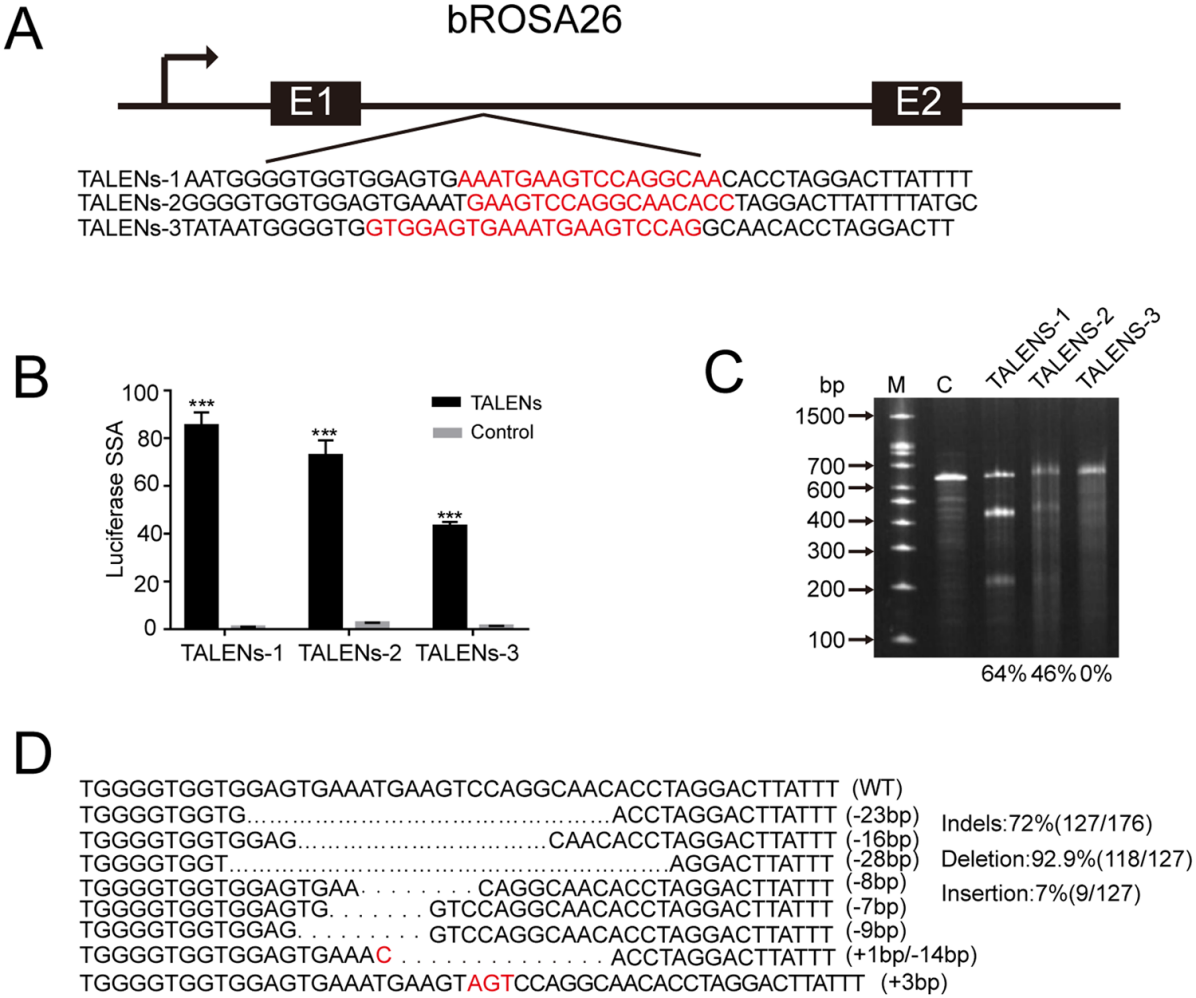
Assessment of TALEN activity. (**A**) Schematic of the bRosa26 targeting locus. The lower panel shows the sequences of the three pairs of TALENs binding to specific genomic sites. The DNA sequence of the primary binding site for each TALEN is shown in black. The sites cleaved by dimerization of the FokI nuclease domains are shown in red. (**B**) The cleavage activity of each TALEN in human 293 T cells was measured using a luciferase SSA assay. Data are presented as the means ± SD from three independent experiments. ***P < 0.001. (**C**) The targeted locus was PCR amplified, and cleavage of the locus was measured using a T7E1 assay. The degree of cleavage was quantified and is shown below each lane. (**D**) Some representative sequences revealed distinct TALEN-induced insertions and deletions at the targeted locus.

TALENs efficiently mediate the knock-in of a reporter vector into the pRosa26 locus. Therefore, we selected the intron of the bRosa26 locus for targeting with three pairs of TALENs (Fig. 2A). First, the activities of TALENs in human 293 T cells were screened with a luciferase single-strand annealing (SSA) assay^24^. All three pairs of TALENs displayed cleavage activity (Fig. 2B). Subsequently, a T7 endonuclease 1 (T7EI) assay was used to assess the frequencies of TALEN-induced indels in bovine fetal fibroblasts (BFFs). TALEN pairs were transfected into the BFFs using nucleofection. After 72 h, the genome of the BFFs was extracted and analyzed. TALEN pair 1 cleaved the target site with the greatest efficiency, as evidenced by the increased incidence of allelic mutations (non-homologous end joining (NHEJ) frequency) (Fig. 2C). TA-cloning and a DNA sequencing analysis of the PCR amplicons corroborated the induction of TALEN-mediated mutations at the endogenous site (Fig. 2D). Therefore, we used TALEN pair 1 in subsequent experiments.

### Efficient gene knock-in at the bovine Rosa26 locus

**Figure 3:**
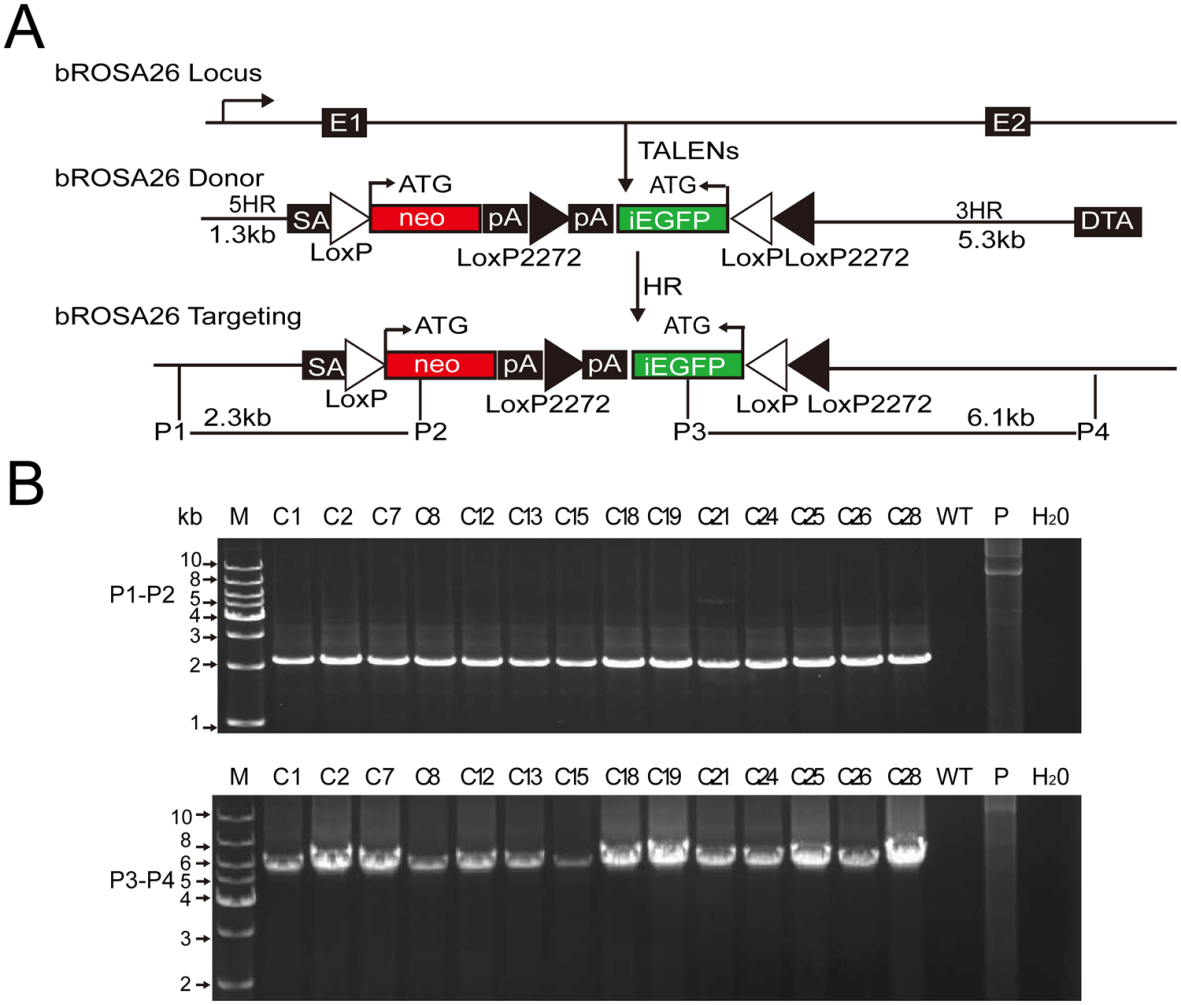
Production of bRosa26-iEGFP cell clones. (**A**) A diagram for TALEN-mediated knock-in of bRosa26-iEGFP into the bRosa26 locus. White triangles indicate WT loxP sites. Black triangles indicate loxP2272 sites, and SA indicates the splice acceptor. (**B**) PCR analysis of the positive gene knock-in cell clones. Primers P1 and P2 amplified a 2.3 kb product, confirming the production of positive bRosa26-iEGFP knock-in cell clone. Primers P3 and P4 amplified a 6.1 kb product and confirmed the production of positive bRosa26-iEGFP knock-in cell clone. M, 1 kb DNA ladder; Lanes 2–15, positive cell clones; WT, wild-type BFFs; P, donor vector; H_2_O, negative control.

A reporter donor vector was constructed, which consisted of a splice acceptor (SA), a promoterless neomycin (neo) resistance gene, and an inverted EGFP (iEGFP) flanked by homologous arms, to determine whether the bRosa26 region enabled the ubiquitous expression of an inserted gene, similar to the Rosa26 locus in other species. The loxP and mutant loxP2272 sites, which flanked the neo and iEGFP genes, respectively, were used for Cre-mediated removal of the neo gene and inversion of the iEGFP gene. As a result, EGFP will be activated to express under the control of the bRosa26 promoter (Fig. 3A). Moreover, these sites allow virtually any gene of interest to be inserted into the bRosa26 locus using RMCE. For gene targeting, BFFs were electroporated with the linearized targeting vector accompanied by TALEN pairs. After selection with G418 (1 mg/ml from day 7 to day 10), 37 cell clones were screened and expanded (Table 1). Twenty-eight of the 37 clones (bRosa26-iEGFP) were correctly targeted, based on PCR analyses of the 5′- and 3′-arms. The PCR results of several positive clones are shown in Fig. 3B. The positive bRosa26-iEGFP cell clones were used in subsequent experiments.

**Table 1:** Targeting efficiency of bRosa26-iEGFP.

Cell line	Screening method	Isolated colonies	Positive cell colonies	Targeting efficiency (%)	Cell colonies for NT
1003	G418	28	37	75.8% (28/37)	#7

### Transgenes at the bRosa26 locus are expressed ubiquitously

**Figure 4:**
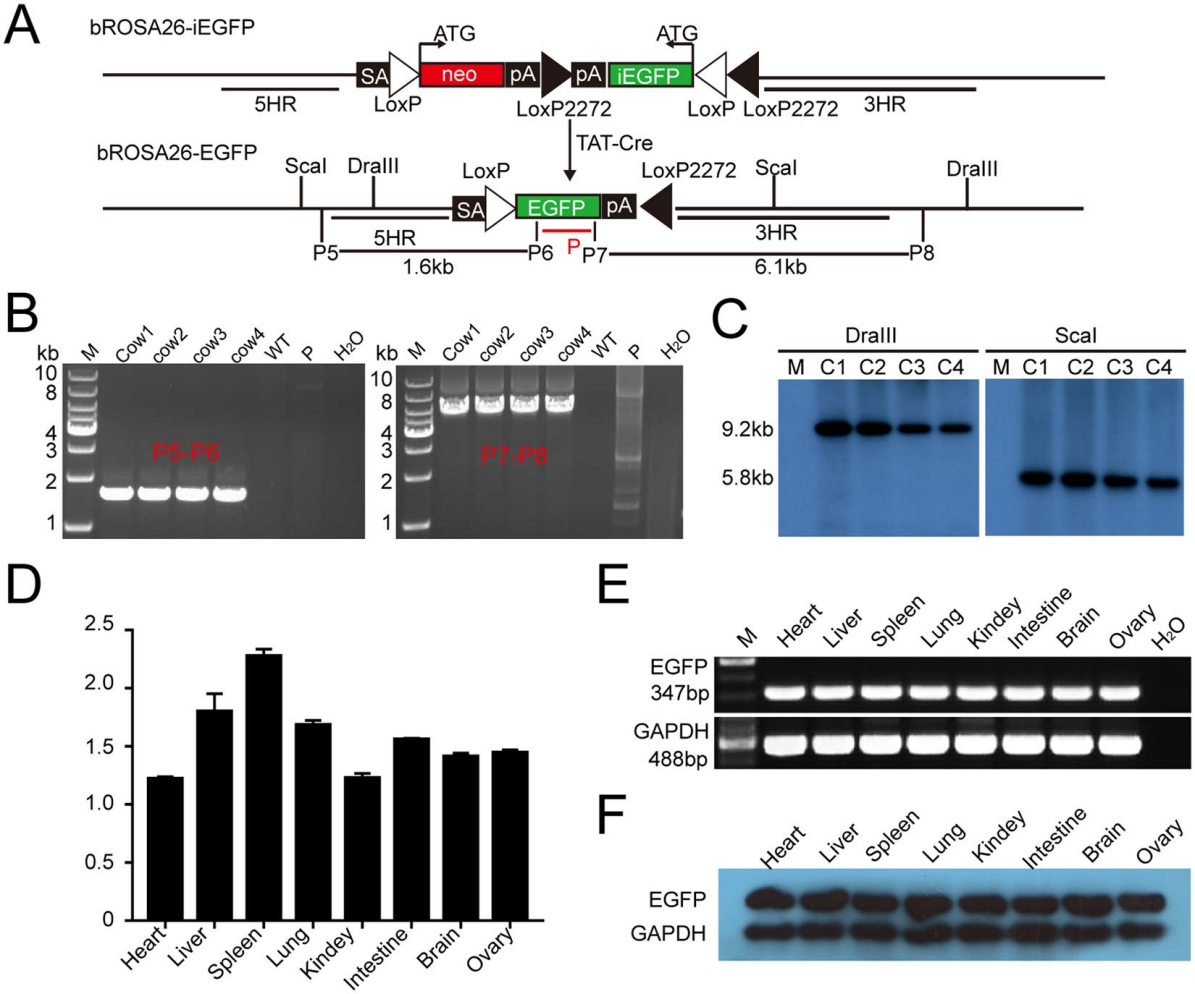
Profiles of transgene expression from the bRosa26 locus. (**A**) TAT-Cre-mediated recombination induces EGFP expression. (**B**) PCR analysis of transgenic cloned cattle. Primers P5 and P6 amplified a 1.6 kb product, confirming the production of positive bRosa26-EGFP knock-in cloned cattle. Primers P7 and P8 amplified a 6.1 kb product and confirmed the production of positive bRosa26-EGFP knock-in cloned cattle. M, 1 kb DNA ladder; Lanes 2–5, genomic DNA from bRosa26-EGFP knock-in cloned cattle; P, donor vector; H2O and WT, negative controls. (**C**) Southern blot identification of transgenic cloned cattle. Upon digestion with DraIII or ScaI, a band of 9.2 kb or 5.8 kb was detected in transgenic cloned cattle, respectively. C1-C4, genomic DNA from bRosa26-EGFP knock-in cloned cattle; WT, wild-type cattle. (**D**) EGFP expression in various tissues from bRosa26-EGFP cattle relative to GAPDH expression, as determined by Q-PCR. Error bars represent the mean ± SD. (**E**) EGFP expression in various tissues from the bRosa26-EGFP cattle, as determined by RT-PCR. For RT-PCR, the designed primers annealed to the EGFP and amplified a correctly spliced product of 347 bp. GAPDH served as a control (488 bp). (**F**) EGFP expression in various tissues from the bRosa26-EGFP cattle, as determined by Western blot, GAPDH served as a control.

We generated bRosa26-EGFP cells by adding the TAT-Cre protein to bRosa26-iEGFP cell line #7 to determine whether the bRosa26 promoter ubiquitously drives gene expression similar to the Rosa26 promoter of other species (Fig. 4A). TAT-Cre proteins were purified from a bacterial source using a two-step method based on our previously study^21,22^ (Supplementary Fig. S2A and B) and were then used to analyze the activity in an *in vitro* recombination reaction. We constructed a pDFR plasmid, which was used as a substrate (Supplementary Fig. S2C). Incubation of linearized pDFR (8.3 kb) with Cre resulted in a linearized pL (5.7 kb) and a recircularized pC (2.0 kb). Based on the results of the *in vitro* assay, the TAT-Cre protein functioned to recombine the substrate (linearized pDFR, 8.3 kb in size) and produced two bands (2.0 kb and 5.7 kb in size) (Supplementary Fig. S2D). Subsequently, we used the TAT-Cre protein to treat bRosa26-iEGFP cell line #7 and obtain bRosa26-EGFP cells (Supplementary Fig. S2E and F), which were used as donors to perform nuclear transfer (NT). As shown in Supplementary Fig. S3A, EGFP was ubiquitously expressed from the 2-cell stage to the blastocyst stage. The pregnancy of a surrogate was terminated 46 days after embryo transfer, and one fetus was obtained. EGFP was ubiquitously expressed in all organs of the fetus (Supplementary Fig. S3B and C). As shown in Table 2, we obtained four bRosa26-EGFP cloned cattle. However, four of them died soon after birth due to the commonly observed effect of somatic cell cloning or because these four cattle were twins. PCR and Southern blot analyses showed that all four cattle were derived from the donor cells obtained after somatic cell nuclear transfer (SCNT) (Fig. 4B,C). In the positive bRosa26-EGFP cloned cattle, EGFP was ubiquitously expressed in all organs examined in this study (Supplementary Fig. S4A). In addition, Q-PCR, RT-PCR and Western blotting showed the ubiquitous expression of EGFP in all organs examined in this study (Fig. 4D–F and Supplementary Fig. S4B,C). Thus, the bRosa26 locus is an excellent site for inducing the ubiquitous expression of exogenous genes.

**Table 2:** Summary of NT results.

No. of the cell clone	Oocytes	Reconstructedembryos	Blastocysts	Blastocysts	Recipients	Pregnancy at day 60	Cattle born (deaths)
#7	560	416	75	18.0%	8	2	4 (4)

### RMCE at the bRosa26 locus mediated by TAT-Cre

**Figure 5:**
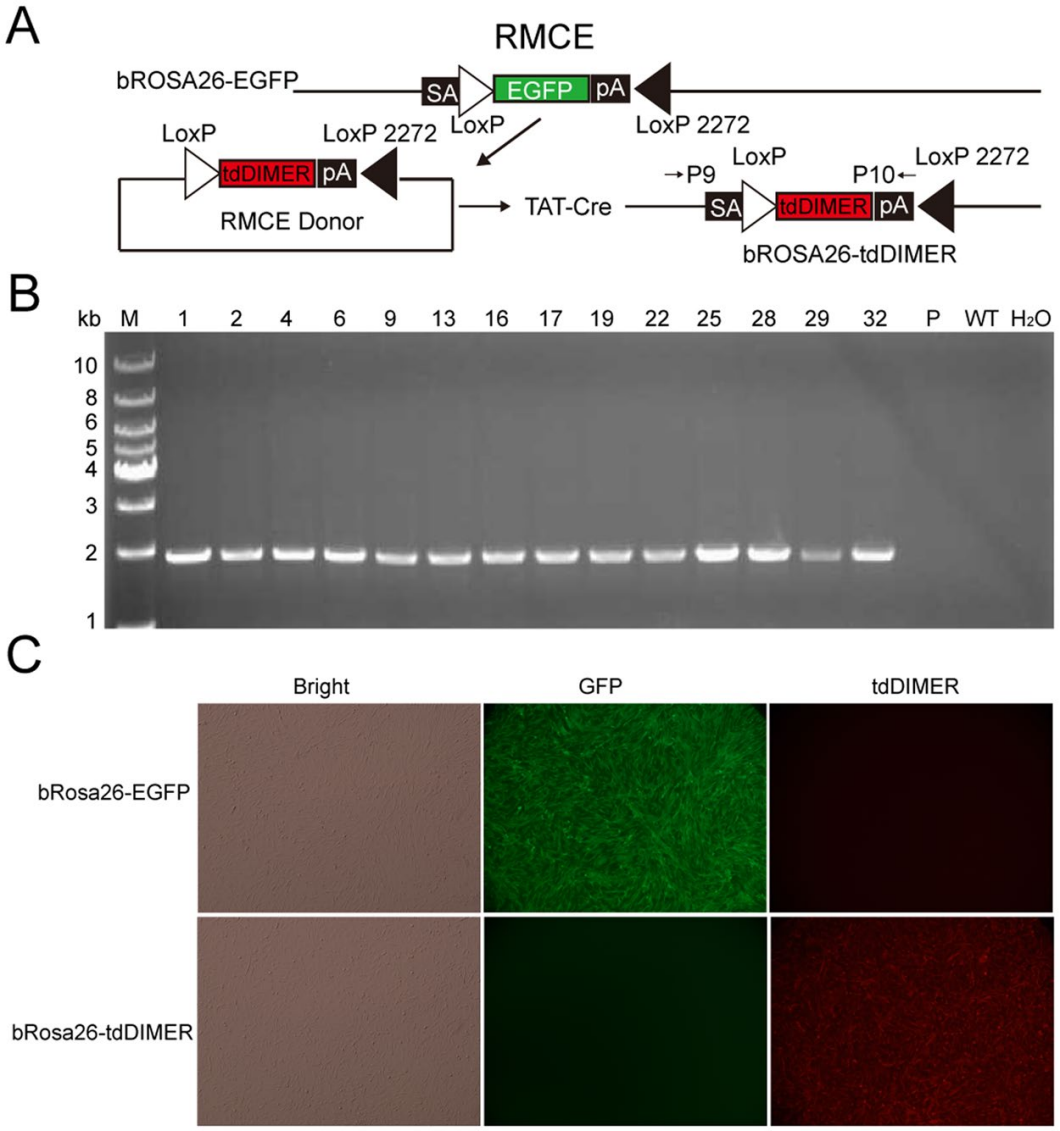
TAT-Cre mediated RMCE in bRosa26-EGFP reporter cells. (**A**) RMCE replaces EGFP with tdimer2(12). (**B**) PCR analysis of bRosa26-tdimer2(12) cell clones. Primers P9 and P10 amplified a 1.9 kb product and confirmed the production of positive RMCE cell clones. M, 1 kb DNA ladder; Lanes 2–15, genomic DNA from bRosa26-tdDIMER cell clones; P, RMCE donor vector; H_2_O and WT, negative controls. (**C**) Fluorescence microscopy images showing the detection of the bRosa26-EGFP and bRosa26-tdDIMER cells.

RMCE at a “safe locus” is important for creating farm animal transgenics because it avoids random integration and does not require drug selection. The insertion of heterotypic loxP sites along with EGFP into bRosa26 introduced a donor site, which allowed us to replace the EGFP cassette in the bRosa26 locus with any gene of interest by RMCE. We engineered an exchange vector (Fig. 5A) containing a promoterless tandem Dimer of DsRed (tdimer2(12))^25^ flanked by heterotypic loxP sites to test the feasibility of this approach. This exchange vector named bRosa26-tdDIMER was electroporated into bRosa26-EGFP fetal fibroblasts (Fig. 5A). The recombinant TAT-Cre protein was used to avoid integration of the Cre plasmid. After screening single-cell cultures, we isolated 88 colonies. The PCR analysis confirmed the successful replacement of EGFP with tdimer2(12) in 43 colonies (Table 3). Partial PCR results are shown in Fig. 5B. The positive bRosa26-tdDIMER cell clone was also identified by fluorescence microscopy (Fig. 5C). As expected, tdimer2(12) was ubiquitously expressed in the cell clones. Based on these results, the TAT-Cre-mediated RMCE technology can be used in cattle along with our bRosa26-EGFP reporter cell line.

**Table 3:** RMCE efficiency of bRosa26-EGFP.

Cell line	Screening Method	Isolated colonies	Positive cell colonies	Targeting efficiency (%)
1701	Single-cell culture	88	43	48.9%

## Discussion

Precision genetic engineering based on the stable chromosomal insertion of exogenous DNA at a “safe locus” of the bovine genome may become important for the development of improved biomedical models, pharmaceutical research and accelerated breeding programs. Although this approach has been widely established in mice^9,26^, rats^16^, human cells^10,15^ and pigs^17^, this technology has been poorly investigated in bovine. The bRosa26 locus characterized here is the first transgene safe harbor identified in bovine. Similar to mice^12,27^, rats^16^, humans^15^, pigs^17–19^, and rabbits^20^, this locus consists of highly conserved promoter and exon 1 sequences, as well as the location of a flanking gene. Meanwhile, the bRosa26 locus comprises two exons and encodes lncRNAs, which were also expressed in all tissues examined in the present study.

Efficient gene targeting is an important factor if bRosa26 is to be used as a general purpose permissive locus for transgene placement, similar to mouse Rosa26. The bRosa26 locus was highly amenable for TALEN-mediated knock-in in the present study, with greater than 70% efficiency for the bovine fetal fibroblasts. Hence, the application of the transgene safe-harbor locus identified in this study in combination with emerging gene-editing nuclease tools, such as CRISPR/Cas9^28,29^, may enable the efficient and simple targeted integration of a single copy (or any desired number) of a transgene with predictable transgene expression in the future. We confirmed that a foreign reporter gene inserted into the bRosa26 locus was expressed ubiquitously in various stages, including cells, embryos, fetus and cattle. Strategies targeting the bRosa26 locus may overcome the problem of gene silencing and the concerns regarding gene disruptions or alterations induced by insertional mutagenesis.

RMCE is a simple and effective technique for replacing and expressing any gene at a safe locus^14^. Recently, RMCE has also been used in transgenic pigs^17^, but it has not been reported in bovine. Therefore, we created a valuable master bRosa26-EGFP fetal fibroblast cell line in which any genetic material of interest can be efficiently introduced and stably overexpressed using RMCE without the need for a drug-resistance gene. We used the TAT-Cre protein^30^ in bovine fetal fibroblasts for the first time to perform RMCE and avoid integration of the Cre plasmid. These features define bRosa26 as a safe-harbor locus that is attractive for versatile transgenic applications, including gain-of function, loss-of-function and lineage-tracing studies in bovine.

Many normal genetically modified animals produced using the Rosa26 safe locus live. However, the four cloned cattle all died soon after birth in our study, which may have been due to several factors. First, SCNT is associated with increased rates of abortion and health problems, such as death soon after birth, due to incomplete epigenetic reprogramming of the somatic donor nuclei^31,32^. Therefore, many reconstructed embryos must be transplanted into a large number of recipient animals. Only eight recipient animals were used in our study, which was fewer than the number employed in our previous study^5,33^. Second, the cattle is a singleton animal, but we transplanted two embryos, resulting in twins and a higher chance of death. The zygote injection pathway has been used to efficiently generate random insertions, deletions (indels) or knock-in sequences in cattle. These studies provide strong technological support for the use of bRosa26 locus in the future.

In summary, the bRosa26 locus characterized here is the first transgene safe-harbor locus identified in the bovine genome. We confirmed that foreign genes inserted into the bRosa26 locus were expressed ubiquitously, and we created a valuable master bRosa26-EGFP fetal fibroblast cell line in which any genetic material of interest can be efficiently introduced and stably overexpressed using RMCE. Hence, the results of this study will serve as an excellent platform for generating genetically modified cattle for biomedical and agricultural applications, particularly the application of the transgenic cattle mammary bioreactor.

## Materials and Methods

### Identification of the bRosa26 locus

The mouse Rosa26 promoter and exon 1 sequences were used as a template to search the Ensembl bovine database. A highly conserved sequence (sequence similarity of 83%) on bovine chromosome 22 was identified as the putative bRosa26 locus. The sequence of bRosa26 exon 1 was predicted using the alignment of mouse, rat, human, and pig Rosa26 exon 1 sequences.

### 3′ RACE and 5′ RACE analyses

We performed 3′ RACE and 5′ RACE analyses using the SMARTer RACE cDNA Amplification Kit (Clontech, Palo Alto, CA, USA). According to the manufacturer’s protocol, the cDNA templates used for 3′ RACE and 5′5′ RACE were synthesized using 5′-CDS Primer A and 3′-CDS Primer A, respectively. PCR was conducted with the universal primer mix (UPM) and 3′ or 5′ gene-specific primers (GSPs; 3′-GSP, 5′-GGCTCCTCAGAGAGCCTCGGC-3′; 5′-GSP, 5′- GCACAGCCTCTTCTCTAGGTGG -3′). The PCR program consisted of 35 cycles at 94 °C for 30 s, 58 °C for 30 s, and 72 °C for 20 s with a final hold at 72 °C for 10 min. PCR products were cloned into PMD19T (TaKaRa, Kusatsu, Shiga, Japan) for sequencing.

### RT-PCR and Q-PCR analyses

Total mRNAs were extracted from various adult tissues using Trizol Reagent (Thermo Fisher Scientific, Waltham, MA, USA) prior to performing RT-PCR and Q-PCR analyses. The cDNA templates were synthesized using a FastQuant RT Kit (Tiangen, China), and the genomic DNA was then digested with DNase I. Primer R (R, 5′-AGCCTGCTTTGTCACCCTCAT-3′), located in exon II, was used to perform RT-PCR along with Primer F2 (F2, 5′-GGCTCCTCAGAGAGCCTCGGC-3′) (Supplementary Fig. S1C). The bovine glyceraldehyde-3-phosphate dehydrogenase (GAPDH) gene was used as the control gene (5′-GCAAGTTCCACGGCACAG-3′ and 5′-CGCCAGTAGAAGCAGGGAT-3′). The expected PCR products for the bRosa26 lncRNA and GAPDH were 155 bp and 488 bp, respectively. Quantitative real-time PCR was performed using Power SYBR Green Mix (Roche, Mannheim, Germany). Primers with sequences of 5′-AGCCTGCTTTGTCACCCTCAT-3′ and 5′-GGCTCCTCAGAGAGCCTCGG-3′ were used to detect bRosa26 ncRNA expression by amplifying an exon II fragment. Primers with sequences of 5′-GAACCGCATCGAGCTGAA-3′ and 5′-TGCTTGTCGGCCATGATATAG-3′ were used to detect EGFP. Primers with sequences of 5′-CATGTTTGTGATGGGCGTG-3′ and 5′- CATCGTGGAGGGACTTATGAC-3′ were used to amplify bovine GAPDH as the reference.

### Construction of vectors

These vectors were constructed using standard molecular cloning methods. The pbRosa26-iEGFP donor vector consisted of the 1.3 kb 5′ homology arm and the 5.3 kb 3′ homology arm flanking an adenoviral splice acceptor sequence, a promoterless neo gene and an inverted EGFP (iEGFP) gene were inserted between the homologous arms. The loxP and loxP2272 sites were arranged to flank the neo and iEGFP genes. The bRosa26-tdDIMER vector consisted of the tdimer2(12) cassette with a SV40 polyA signal sequence, loxP and loxP2272 sites was amplified from pCMV-Brainbow-2.1 R (Addgene, Plasmid #18723) and contained additional NotI and XhoI enzyme sites, primers sequences are as follows: Forward, 5′-ATGATCCCCAACCTCGCGGCCGCTCGACTGCAGAATTTCGAGA-3′; Reverse, 5′-AAGGATGCAAGAATTCATAACTTCGTATAGGATACTTTATACGAAGTTATAGCGGCTAGATCATAATCAGC-3′. The tdimer2(12) cassette was subcloned into the ploxp_2_ neoDTA at the NotI and XhoI sites. TALEN expression constructs were assembled by the Viewsolid Biotech Company (Beijing, China). Three pairs of TALENs were designed to target intron 1. The TALEN recognition sequences were: left TALEN1 5′-AATGGGGTGGTGGAGTG-3′ and right TALEN1 5′-AAAATAAGTCCTAGGTG-3′, 17 bp spacer AAATGAAGTCCAGGCAA; left TALEN2 5′-GGGGTGGTGGAGTGAAAT-3′ and right TALEN2 5′-GCATAAAATAAGTCCTA-3′, 17 bp spacer GAAGTCCAGGCAACACC; and left TALEN3 5′-TATAATGGGGTGGTGGA-3′ and right TALEN3 5′-AAGTCCTAGGTGTTGC-3′, 16 bp spacer GTGAAATGAAGTCCAG.

### Luciferase single-strand annealing (SSA) assay

The luciferase reporter plasmids were constructed using the SSA Kit (Viewsolid Biotech, Beijing, China) based on described previously^24^. The luciferase gene contained two repeats that was disrupted by a “stop” signal. Effective TALEN pairs would generate a double-strand break (DSB) in the “stop” sequence, which allow the functional luciferase gene to be restored. The TALEN pairs plasmids (500 ng each) and luciferase reporter plasmid (100 ng) were co-transfected into human 293 T cells in 48-well plate using Lipofectamine 3000 (Invitrogen, USA). After 48 h, the cells were lysed and luciferase activity was determined using luciferase assay reagent (Promega, USA).

### T7 endonuclease 1 (T7EI) assay

TALEN pairs were transfected into the BFFs using nucleofection. After 72 h, the genome of the BFFs was extracted and the editing activity of each TALEN was assayed using T7 endonuclease I (T7E1) (New England Biolabs, USA), as described previously^34^. Briefly, genomic DNA was extracted from TALEN-treated cells using a DNeasy Blood and Tissue kit (Qiagen, Hilden, Germany). PCR amplicons including nuclease target sites were generated using the primers: Rosa26-F, 5′-GCCGCAATACCTTTATGGGAG-3′ and Rosa26-R, 5′-ATTGGTGGTGAAACCTGTCTG-3′. The 700 bp PCR amplicons were denatured by heating and annealed to form heteroduplex DNA using a thermocycler and then digested with T7E1 for 30 min at 37 °C and then analyzed using agarose gel electrophoresis. Mutation frequencies (indels, %) were calculated by quantifying the relative using ImageJ software.

### Cell culture and transfection

Primary BFFs were isolated from a Holstein cattle fetus by disaggregating the entire body, with the exception of the head and viscera, and cultured in Dulbecco’s Modified Eagle’s Medium (DMEM; Gibco, Grand Island, New York, USA) supplemented with 10% fetal bovine serum (FBS) (Gibco, Grand Island, New York, USA) at 37.5 °C in an atmosphere of 5% CO_2_ and humidified air. Next, 4 µg of TALEN pair 1 and 4 µg of linearized pbRosa26-iEGFP donor were nucleofected into 1 × 10^6^ BFFs using Amaxa Nucleofector reagent (Lonza Group AG Basel, Switzerland) according to the manufacturer’s guidelines and the program T-016. G418 (1 mg/ml) selection was used in cell colonies that formed within 48 h after transfection, and the cell density was approximately 1 × 10^5^ cells/dish (10 cm^2^). Individual cell clones were isolated 7–10 days after G418 selection, expanded, cultured, sequenced and cryopreserved after a total of 12–14 days in culture.

### Purifying the TAT-Cre protein

The pTAT-Cre vector was purchased from Addgene (plasmid #35619). The TAT-Cre protein was produced *in vitro* by transfecting the BL21 (DE3) E. coli strain with the pTAT-Cre vector according to our previously published method^21,22^. Protein expression was induced with 0.5 mM IPTG for 16 h at 16 °C. TAT-Cre was purified using Ni-NTA resin (Qiagen, Hilden, Germany) and cation-exchange chromatography (HiTrap SP HP) (GE Healthcare, Uppsala, Sweden), according to the manufacturer’s manual. The protein purity was examined by SDS-PAGE. The protein concentration was determined with a Bradford assay and adjusted to approximately 1 µg/µl. Proteins were stored at −80 °C.

### Generation of bRosa26-EGFP master cell lines

Approximately 1.0 × 10^5^ bRosa26-iEGFP cells were plated on 48-well cell culture plates and cultivated. After 24 h, cells were treated with 2 µM recombinant TAT-Cre proteins in serum-free DMEM (Gibco, Grand Island, New York, USA) for 2 h and then washed and cultivated for an additional 48 h with DMEM supplemented with 10% FBS (Gibco, Grand Island, New York, USA). Cells expressing EGFP were used to perform rejuvenation based on a previous method. Briefly, E46 embryos of bRosa26-EGFP fetuses from which the head, limbs, and internal organs had been removed were minced and digested with 0.25% Trypsin-EDTA in DMEM supplemented with 10% FBS (Gibco, Grand Island, New York, USA) and 1% penicillin-streptomycin (Gibco, Grand Island, New York, USA) for 10 min at 37 °C. Dissociated cells were centrifuged at 1000 g for 5 min and cultivated in DMEM containing 10% FBS on 10 cm^2^ culture dishes. Cells were frozen in a CellBanker 2 (ZENOAQ, Tokyo, Japan) for future use.

### Production of cloned embryos and cattle

The somatic cell nuclear transfer (SCNT) procedure was performed as previously described^35^. Briefly, the nuclei of transgenic cells were transferred into enucleated oocytes to produce reconstructed embryos *in vitro* using the ECM® 2001 Electro Cell Manipulation System (BTX, San Diego, CA, USA). Day 7 blastocysts were collected for future transplantation. Sixteen blastocysts were transferred into 8 recipient cattles. One to two transgenic cloned blastocysts were transferred into each recipient. Pregnancy was detected by ultrasonography at 60 days and 180 days post-transfer. All experiments were performed in accordance with the relevant guidelines and regulations, and the Institutional Animal Care and Use Committee of China Agricultural University approved this research.

### Southern blot

Genomic DNA was extracted from animal ear tissue using phenol/chloroform. At least 10 μg of genomic DNA from transgenic and wild-type cattle were digested with the ScaI or DraIII restriction enzyme (New England Biolabs, USA) overnight. A 347 bp EGFP probe was amplified with the primer pairs 5′-ATGGTGAGCAAGGGCGAGGAG-3′ and 5′-TTACTTGTACAGCTCGTCCATGC-3′ and was labeled using a PCR DIG Probe Synthesis Kit (Roche, Mannheim, Germany). After electrophoresis on a 0.8% agarose gel for 6 h, the DNA was transferred to a nitrocellulose membrane (Roche, Mannheim, Germany). Pre-hybridization and hybridization were performed at 45 °C, and washing steps were performed at 68 °C. The positive bands were expected to be 5.8 kb and 9.2 kb, respectively.

### Western blotting

Samples were isolated from different tissues from the transgenic and wild-type (WT) cattles and homogenized in **c**ell lysis buffer for Western and IP analyses (Beyotime, Shanghai, China). After centrifugation at 10000 g for 10 min at 4 °C, the total protein supernatants were collected, and protein concentrations were measured using a BCA Protein Assay kit (Beyotime, Shanghai, China). Approximately 20 μg of protein was separated on 10% SDS-PAGE gels and transferred to Immobilon-P membranes (MilliporeSigma, Burlington, MA, USA). After blocking in 3% BSA in TBST for 1 h, membranes were incubated with a GFP antibody (dilution, 1:10000; Abcam, Cambridge, MA, USA) or bovine GAPDH antibody (dilution, 1:10000; Abcam, Cambridge, MA, USA) overnight at 4 °C. After washes with TBST, membranes were incubated with a goat anti-rabbit antibody conjugated with horseradish peroxidase (dilution, 1:20000; Sino-American Co, Beijing, China) for 1 h followed by three washes with TBST. Protein signals were detected using an ECL Chemiluminescence kit (Thermo Fisher Scientific, Waltham, MA, USA).

### RMCE for replacing EGFP with tdimer2(12) in the bRosa26 locus

bRosa26-EGFP BFFs were electroporated with 5 μg of the bRosa26-tdDIMER plasmid. After 2–4 h, the cells were treated with 2 µM TAT-Cre recombinase in serum-free DMEM (Gibco, Grand Island, New York, USA) for 2 h and were then washed and cultivated for additional 72 h in DMEM containing 10% FBS (Gibco, Grand Island, New York, USA). Cells were then trypsinized and reseeded in plates at a density of approximately 100 cells/dish in cell culture medium without G418 for 6–8 days. Colonies were isolated, transferred to 48-well cell culture plates, and cultured for another 2–3 days. Half of the cells were used for DNA extraction (Qiagen, Hilden, Germany). PCR was performed using primer pair P9 and P10. The PCR program consisted of 35 cycles at 94 °C for 30 s, 58 °C for 30 s, and 72 °C for 2 min with a final hold at 72 °C for 10 min. The expression of the fluorescent protein in positive cell clones was detected by fluorescence microscopy (Olympus BX51).

### Analysis of the expression of the fluorescent protein

The expression of the fluorescent protein in cells, tissues or organs was detected using fluorescence microscopy (Olympus BX51) with appropriate excitation filters.

## Electronic supplementary material

SUPPLEMENTARY INFORMATION
