# Supplementary material for: Efficient targeted integration into the bovine Rosa26 locus using TALENs
Source: Sci Rep. 2018 Jul 10;8:10385. doi: 10.1038/s41598-018-28502-x (PMC6039519; doi:10.1038/s41598-018-28502-x)
Supplement: Supplementary file 1 — SUPPLEMENTARY INFORMATION [file 41598_2018_28502_MOESM1_ESM.docx]

**SUPPLEMENTARY INFORMATION**

**Identification of and efficient gene knock-in at the Rosa26 locus in bovine**

Ming Wang^1,3^, Zhaolin Sun^1,3^, Zhiyuan Zou^1,3^, Fangrong Ding^1^, Ling Li^1^, Haiping Wang^1^, Chunjiang Zhao^2^, Ning Li^1*^_,_ Yunping Dai^1*^


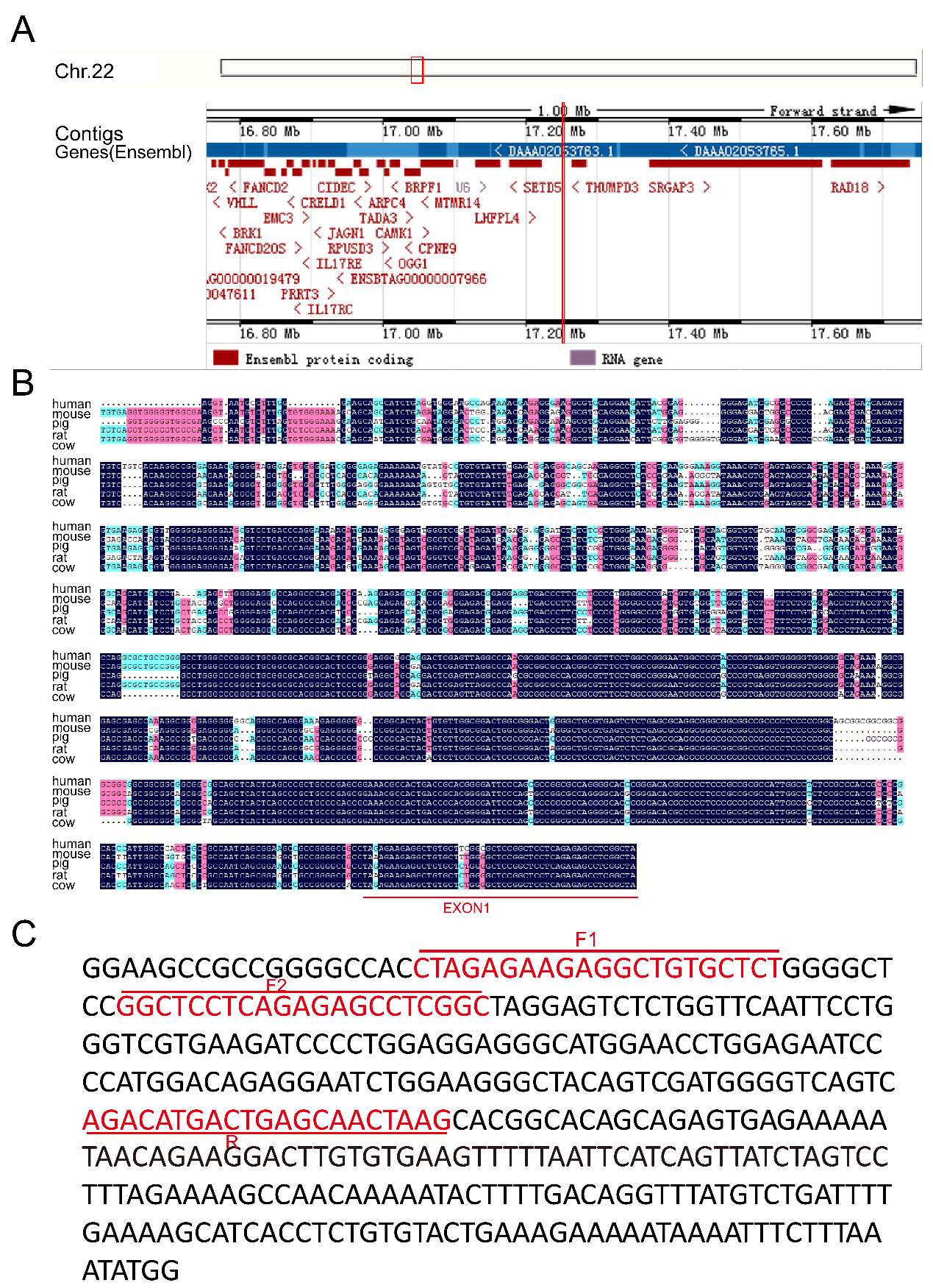


**Supplementary FIG. S1 Identification of the bRosa26 locus and its noncoding RNA**

(A) Diagram depicting bRosa26 loci and the neighboring genes. (B) Comparison of human, mouse, pig, rat and bovine Rosa26 promoter and exon 1 sequences showed high sequence conservation between these species. Rosa26 exon 1 of human, mouse, pig, and rat and the predicted bovine Rosa26 exon 1 are marked with a red line. (C) The sequence of the bRosa26 noncoding RNA. Primer F1 were used in 3’RACE, and Primer F2 and R were used to detect the expression of the noncoding RNA in each tissue.


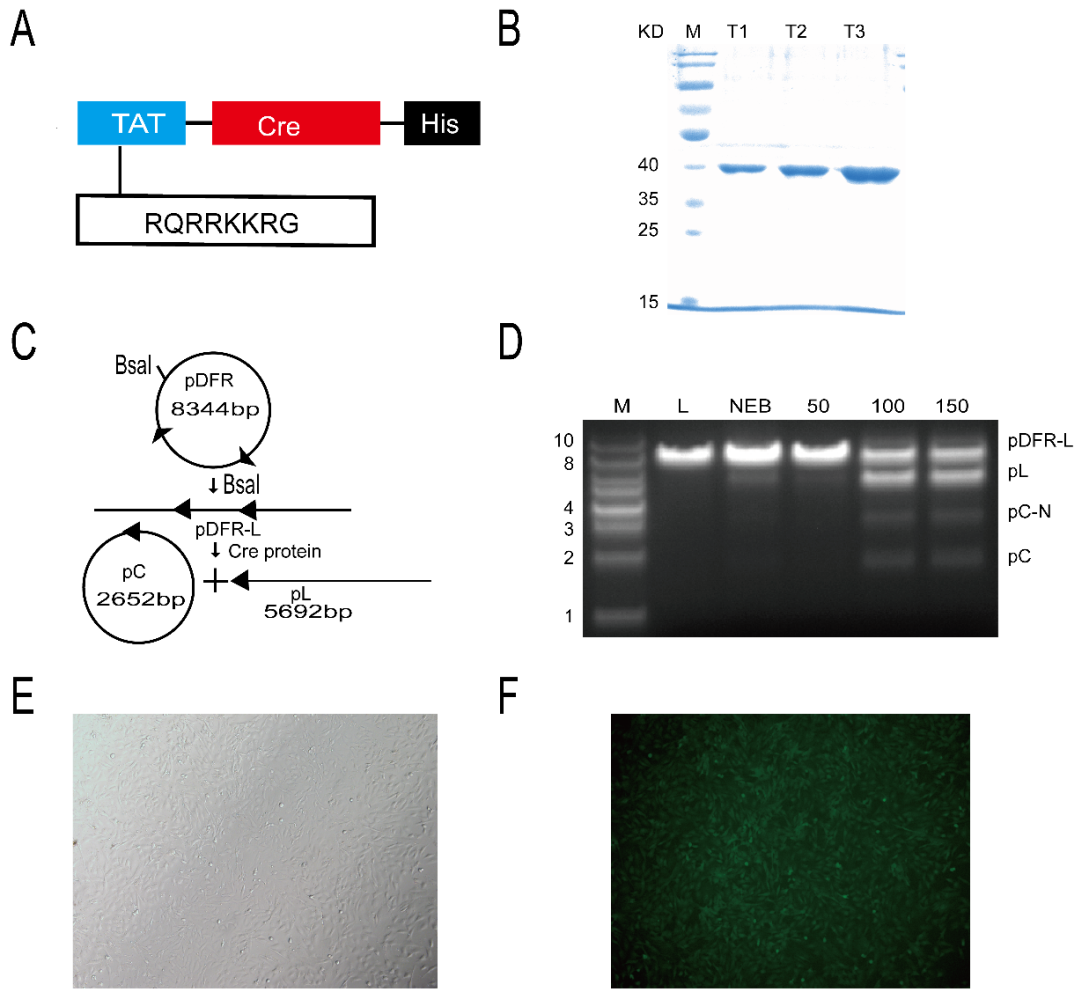


**Supplementary FIG. S2 The generation of the Rosa26-EGFP cells by the TAT-Cre protein**

(A) Schematic description of the TAT-Cre expression constructs that encoded Cre recombinase and a His-tag (represented by red and black boxes, respectively). Blue: TAT (YGRKKRRQRRR). (B) SDS-PAGE analysis of the purification of TAT-Cre proteins. M, marker; T1, 0.5 μg TAT-Cre protein; T2, 1 μg TAT-Cre protein; T3, 3 μg TAT-Cre protein. (C) Schematic of recombination in vitro. The assay substrate, pDFR, was linearized by digestion with BsaI and used to assess the recombinase activity of purified TAT-Cre proteins. (D) Activity of TAT-Cre proteins in vitro. Reactions were carried out in a 50µl volume with 300 ng of pDFR and with NEB-Cre buffer. Different amounts of purified TAT-Cre were added, and the mixtures were incubated at 37 °C for 30 min. The reactions were then split in half and resolved on a 1% agarose gel. Lane 1, 1kb ladder; Lane 2, linearized pDFR; lane 3, NEB Cre; lane 4, 50 ng TAT-Cre; lane 5, 100 ng TAT-Cre; lane 6, 150 ng TAT-Cre. Note the appearance of the recombined circular species (migrating at the 2.0 kb) and the rejoined “stuffer fragment” (5.7 kb). (E) The bRosa26-iEGFP cells that were not treated by the TAT-Cre protein. (F) The bRosa26-EGFP cells that had been treated by the TAT-Cre protein.


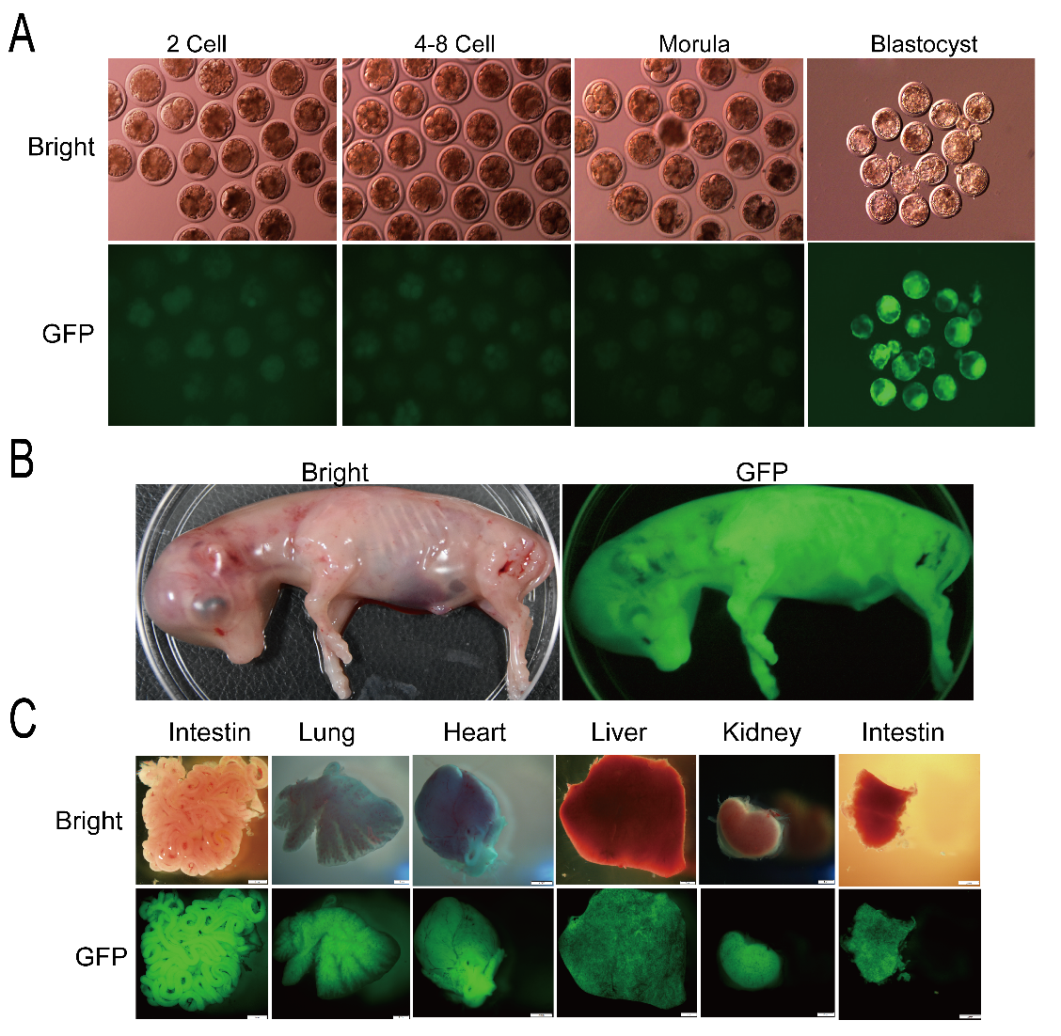


**Supplementary FIG. S3 The EGFP expression profiles at the bRosa26 locus**

(A) EGFP expression of the bRosa26-EGFP cells from the 2-cell stage to the blastocyst stage. (B) Embryonic day E46 bRosa26-EGFP fetus. (C) EGFP expression in various organs in a bRosa26-EGFP fetus.


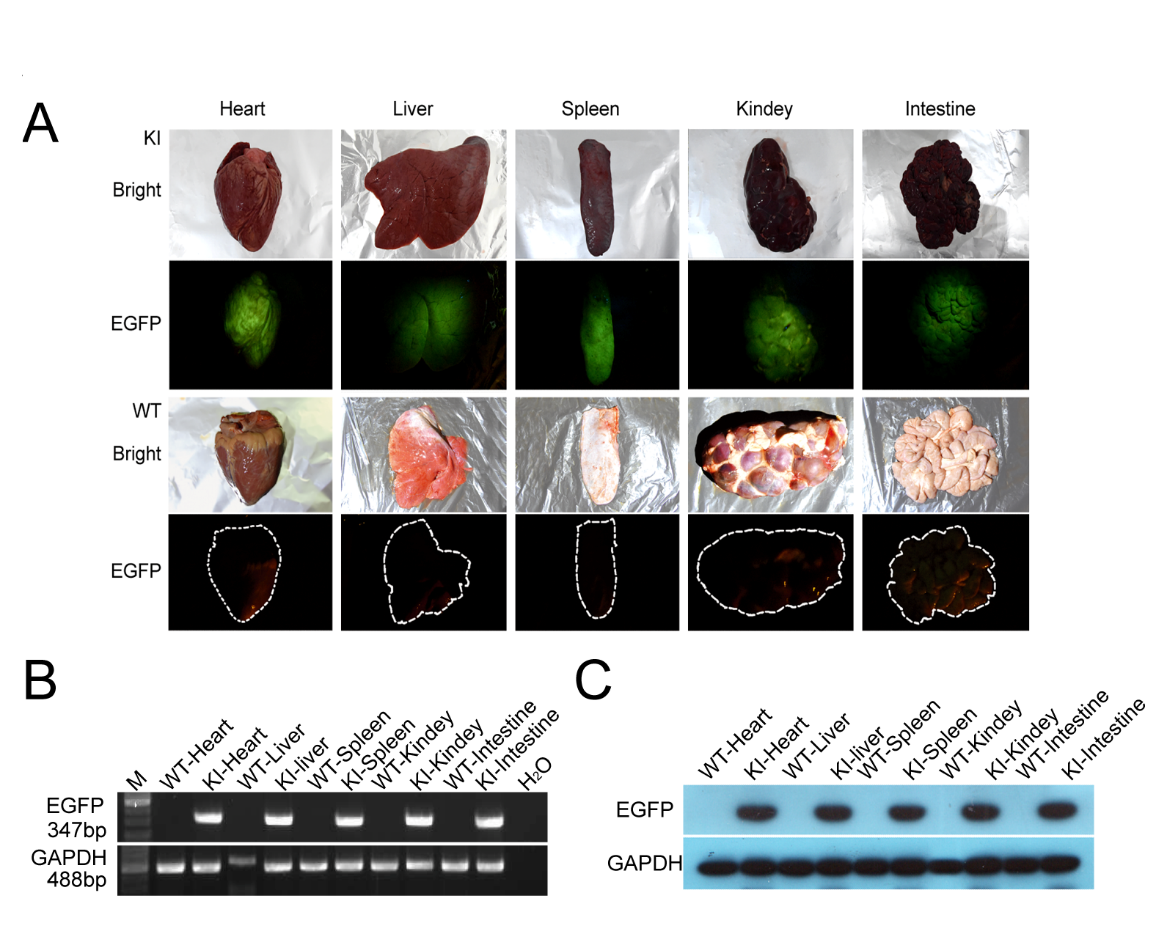


**Supplementary FIG. S4 The EGFP expression in different tissues in bRosa26-EGFP cattle**

(A) The EGFP expression in various tissues was observed using fluorescence microscope. KI: a new-born bROSA26-EGFP cattle, WT: a new-born wild type cattle. (B) The EGFP expression in various tissues relative to GAPDH was analyzed using RT-PCR. For RT-PCR, the designed primers annealed to the EGFP and amplified a correctly spliced product of 347 bp. GAPDH served as a control (488 bp). KI: a new-born bROSA26-EGFP cattle, WT: a new-born wild type cattle (C) The EGFP expression in various tissues was analyzed using Western blot, GAPDH was used as the control. KI: a new-born bROSA26-EGFP cattle, WT: a new-born wild type cattle.


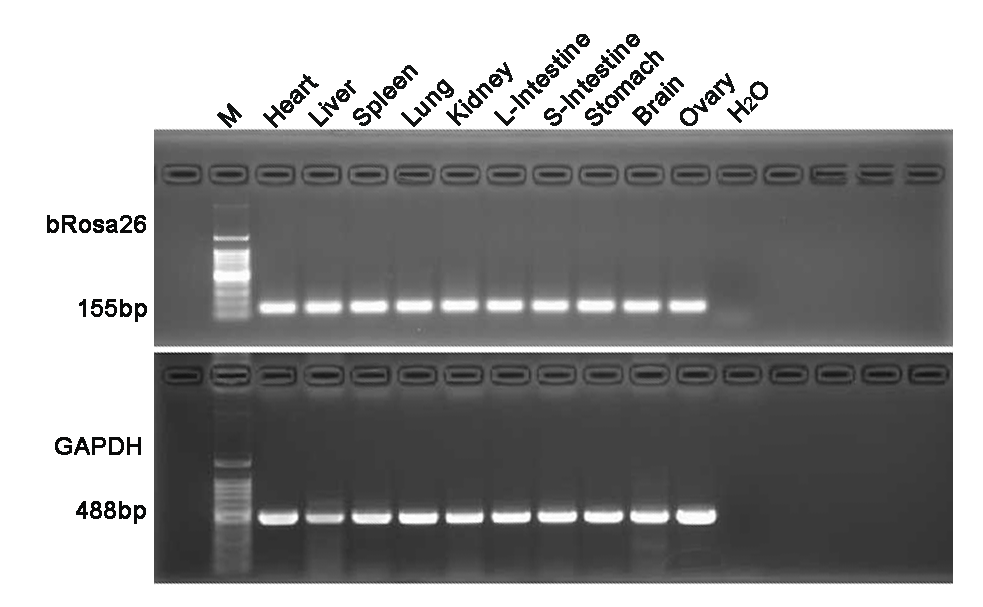


**Supplementary FIG. S5 The whole RT-PCR image of the Fig. 1C**

Expression of bRosa26 lncRNA in various tissues relative to GAPDH by RT-PCR. The designed primers annealed to the bRosa26 sequence and amplified a correctly spliced product of 155 bp, GAPDH served as a control (488 bp).

**
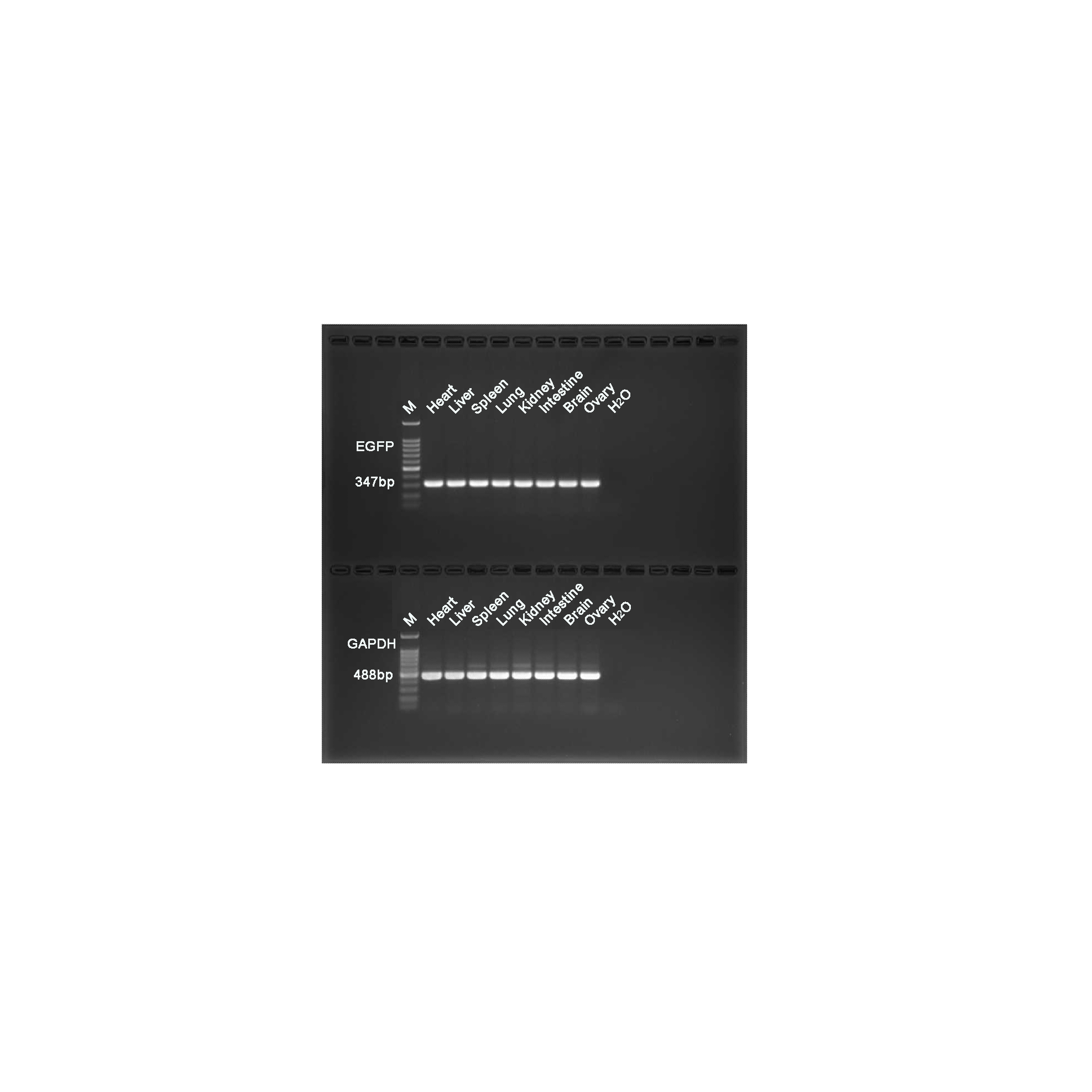
**

**Supplementary FIG. S6 The whole RT-PCR image of the Fig. 1E**

EGFP expression in various tissues from the bRosa26-EGFP cattle, as determined by RT-PCR. For RT-PCR, the designed primers annealed to the EGFP and amplified a correctly spliced product of 347 bp, GAPDH served as a control (488 bp).


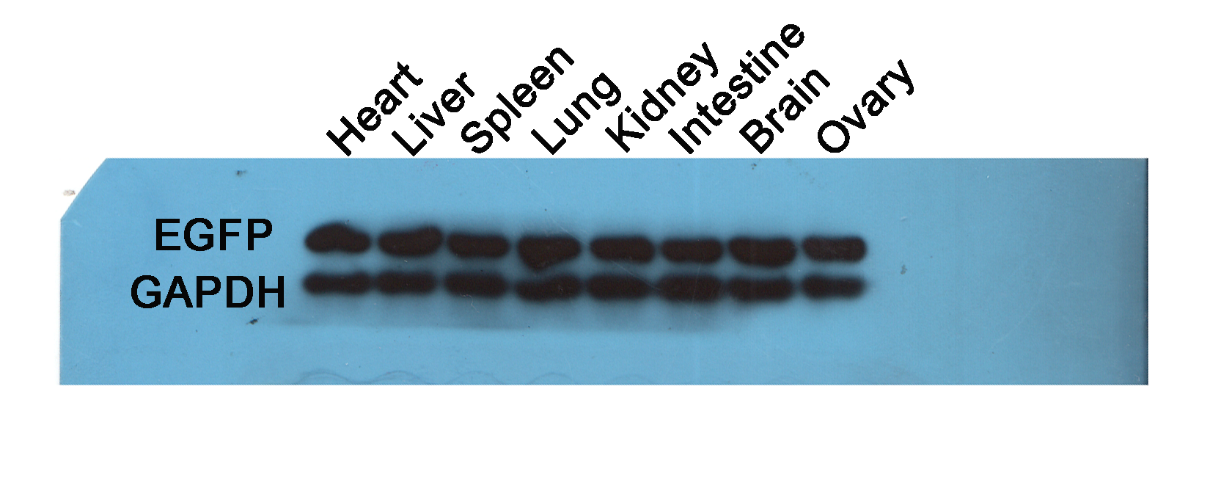


**Supplementary FIG. S7 The whole Western blot image of the Fig. 4F**

EGFP expression in various tissues from the bRosa26-EGFP cattle, as determined by Western blot, GAPDH served as a control.

**
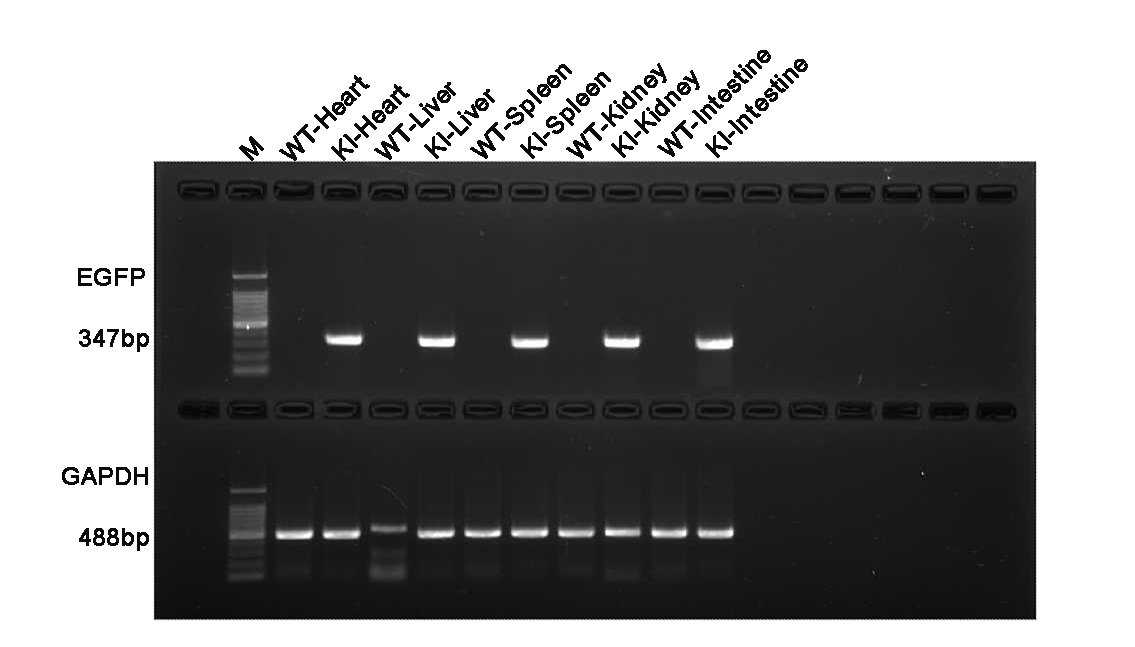
**

**Supplementary FIG. S8 The whole RT-PCR image of the Supplementary FIG. S4B**

The EGFP expression in various tissues relative to GAPDH was analyzed using RT-PCR. For RT-PCR, the designed primers annealed to the EGFP and amplified a correctly spliced product of 347 bp. GAPDH served as a control (488 bp). KI: a new-born bROSA26-EGFP cattle, WT: a new-born wild type cattle.


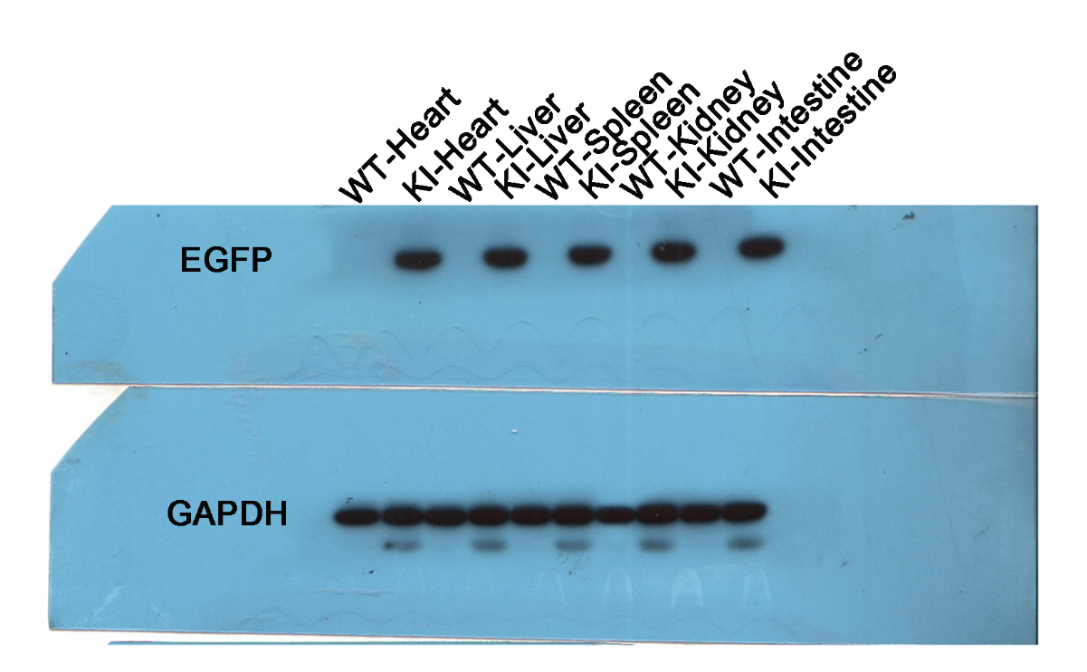


**Supplementary FIG. S9 The whole RT-PCR image of the Supplementary FIG. S4C**

The EGFP expression in various tissues was analyzed using Western blot, GAPDH was used as the control. KI: a new-born bROSA26-EGFP cattle, WT: a new-born wild type cattle.

| PCR Primer | Primer sequence | Applicatiion |
| --- | --- | --- |
| PrimerF1 (3’-GSP) | 5’-CTAGAGAAGAGGCTGTGCTCT-3’ | 3’ RACE gene-specific primers |
| 5’-GSP | 5’-GCACAGCCTCTTCTCTAGGTGG-3’ | 5’ RACE gene-specific primers |
| Primer R | 5’-AGCCTGCTTTGTCACCCTCAT-3’ | RT-PCR、Q-PCR primer for the bRosa26 lncRNA |
| Primer F2 | 5’-GGCTCCTCAGAGAGCCTCGGC-3’ |  |
| GAPDH-F1 | 5’-GCAAGTTCCACGGCACAG-3’ | RT-PCR primer for the control gene GAPDH |
| GAPDH-R1 | 5’-CGCCAGTAGAAGCAGGGAT-3’ |  |
| GAPDH-F2 | 5’-CATGTTTGTGATGGGCGTG-3’ | Q-PCR primer for the control gene GAPDH |
| GAPDH-R2 | 5’-CATCGTGGAGGGACTTATGAC-3’ |  |
| EGFP-F1 | 5’-ATGGTGAGCAAGGGCGAGGAG-3’ | RT-PCR primer for EGFP  PCR primer for EGFP probe |
| EGFP-R1 | 5’-TTACTTGTACAGCTCGTCCATGC-3’ |  |
| EGFP-F2 | 5’-GAACCGCATCGAGCTGAA-3’ | Q-PCR primer for EGFP |
| EGFP-R2 | 5’-TGCTTGTCGGCCATGATATAG-3’ |  |
| Rosa26-F | 5’-GCCGCAATACCTTTATGGGAG-3’ | PCR primer for T7E1 assay |
| Rosa26-R | 5’-ATTGGTGGTGAAACCTGTCTG-3’ |  |
| P1 | 5’- GGGACAGAAAGGCGGAGCGA -3’ | PCR analyses of the 5’ arms for bRosa26-iEGFP cell clone |
| P2 | 5’- AGCCAGTCCCTTCCCGCTTCA -3’ |  |
| P3 | 5’- GAACTTGTGGCCGTTTACGTC -3’ | PCR analyses of the 3’ arms for bRosa26-iEGFP cell clone |
| P4 | 5’- ATGAAACTCTGACCACTACAC -3’ |  |
| P5 | 5’- ACCCATTGGCCAACTCGCT -3’ | PCR analyses of the 5’ arms for bRosa26-EGFP clone cows |
| P6 | 5’- TGAACTTGTGGCCGTTTACGT -3’ |  |
| P7 | 5’- ATATCATGGCCGACAAGCAGA -3’ | PCR analyses of the 3’ arms for bRosa26-EGFP clone cows |
| P8 | 5’- TCTTATTTTTCATCCCACACA -3’ |  |
| P9 | 5’- TAATTATAATGGGGTGGTGGA -3’ | PCR analyses of the RMCE cell clone |
| P10 | 5’- GCCTGCTATTGTCTTCCCAAT -3’ |  |

**Supplementary Table S1 Primers used in this study**
